# 

**Published:** 1890-09

**Authors:** 


### CONTENTS:

Article I.—Some Thoughts on Crown and Bridge-Work. By W. Mitchell, D.D.S. - - - - - 193

" II.—Dental Education. By Dr. J. H. Grant. - - 201

" III.—The Conservative Treatment of the Dental Pulp. By Dr. A. C. Hugenschmidt. - - - 208

" IV.—The new Theory of the Action of Chloroform. By Thomas Gaddes, L.D.S. - - - - 212

### COMMUNICATIONS.

American Academy of Dental Science, - - - - 217

The Dental Protective Association. - - - - - 217

### EDITORIAL.

American Dental Association. - - - - - 219

Identification by the Teeth. - - - - - - 234

### MONTHLY SUMMARY.

Mysteries of the Nerves. - - - - - - - 238

Listerine. - - - - - - - - - 240

